# Genome-Wide Investigation and Functional Analysis of *Sus scrofa* RNA Editing Sites across Eleven Tissues

**DOI:** 10.3390/genes10050327

**Published:** 2019-04-30

**Authors:** Zishuai Wang, Xikang Feng, Zhonglin Tang, Shuai Cheng Li

**Affiliations:** 1 Department of Computer Science, City University of Hong Kong, Kowloon, Hong Kong, China; zishuwang2-c@my.cityu.edu.hk (Z.W.); xikanfeng2-c@my.cityu.edu.hk (X.F.); 2 Department of Pig Genomic Design and Breeding, Agricultural Genome Institute at Shenzhen, Chinese Academy of Agricultural Sciences, Shenzhen 518124, China

**Keywords:** RNA editing, pig, comprehensive analysis, database, tissues

## Abstract

Recently, the prevalence and importance of RNA editing have been illuminated in mammals. However, studies on RNA editing of pigs, a widely used biomedical model animal, are rare. Here we collected RNA sequencing data across 11 tissues and identified more than 490,000 RNA editing sites. We annotated their biological features, detected flank sequence characteristics of A-to-I editing sites and the impact of A-to-I editing on miRNA–mRNA interactions, and identified RNA editing quantitative trait loci (edQTL). *Sus scrofa* RNA editing sites showed high enrichment in repetitive regions with a median editing level as 15.38%. Expectedly, 96.3% of the editing sites located in non-coding regions including intron, 3′ UTRs, intergenic, and gene proximal regions. There were 2233 editing sites located in the coding regions and 980 of them caused missense mutation. Our results indicated that to an A-to-I editing site, the adjacent four nucleotides, two before it and two after it, have a high impact on the editing occurrences. A commonly observed editing motif is CCAGG. We found that 4552 A-to-I RNA editing sites could disturb the original binding efficiencies of miRNAs and 4176 A-to-I RNA editing sites created new potential miRNA target sites. In addition, we performed edQTL analysis and found that 1134 edQTLs that significantly affected the editing levels of 137 RNA editing sites. Finally, we constructed PRESDB, the first pig RNA editing sites database. The site provides necessary functions associated with *Sus scrofa* RNA editing study.

## 1. Introduction

RNA editing is a posttranscriptional regulatory RNA-processing event (excluding RNA splicing) that increases the diversity of transcriptome by changing nucleotides of RNAs. In mammals, the most common form of RNA editing is A-to-I, catalyzed by the adenosine deaminase that acts on RNA (ADAR) family enzymes which contain three members (ADAR1, ADAR2, and ADAR3) [1,2] and the A-to-I editing can lead to an A-to-G reading of the cDNA molecule [3,4]. In mice, knockout of ADAR1 and ADAR2 (or ADARB1) lead to embryonically and postnatally lethal, respectively [5,6] indicating the importance of RNA editing events in normal physiology.

A-to-I RNA editing sites in coding regions that cause synonymous or nonsynonymous mutations have been found within many RNAs, including glutamate receptor subunits [7,8,9], the G protein-coupled serotonin 2C receptor [10] and the antigenome of the hepatitis delta virus [11,12]. Functional consequences of RNA editing in non-coding regions involve miRNA biogenesis [13], editing of miRNA seed regions [14] or target sequences in mRNA [15] and nuclear retention [16]. Moreover, in humans, RNA editing is associated with diseases including autoimmune disorder Aicardi–Goutières syndrome [17], amyotrophic lateral sclerosis [18], autism [19], viral infection [20], and cancer [21].

With the RNA-Seq technology, RNA editing events have been identified in pigs [22,23]. A spatio-temporal editing study in porcine neural tissues showed editing increased during embryonic development concomitantly with an increase in ADAR2 mRNA level, revealing RNA editing may play essential roles in the development of a swine brain [24]. 

Although an increasing number of mammal RNA editing databases have been published [25,26,27,28,29], no database contains the *Sus scrofa* RNA editing sites. The domestic pig (*Sus scrofa*) is a non-rodent animal model used in biomedical research [30,31,32]. In comparison with traditional rodent models, the pig is more similar to humans than mice in body size, growth, development, immunity, physiology, and metabolism, as well as genetically [33,34,35]. Therefore, in this research we constructed the first RNA editing database for *Sus scrofa*. We collected 211 RNA-seq datasets across 11 tissues (blood, brain, fat, heart, kidney, liver, lung, muscle, ovary, spleen, and uterus) from the National Center for Biotechnology Information (NCBI). Then, we identified *Sus scrofa* RNA editing sites and performed statistical analysis. Moreover, we performed molecular properties analyses, including genomic feature annotations and flank sequence characteristics investigation, and potential functional effects, including the impact of A-to-I editing on miRNA–mRNA interactions and RNA editing quantitative trait loci (edQTL) identification.

## 2. Materials and Methods 

### 2.1. Reads Mapping and RNA-Editing Sites Identification

The RNA-seq data were downloaded from the National Center for Biotechnology Information (NCBI) SRA database. The sequence read archive (SRA) ID number is available in Supplemental Table S2. Genomes and annotation files of *Sus scrofa* (Sscrofa11.1) were downloaded from the ENSEMBL database [36]. The reads were mapped to reference genomes using Hisat2 (v2.0.1) [37] with default parameters. The Sequence Alignment/Map Format (SAM) files were sorted and converted to BAM files, the compressed binary version of a SAM file, by Samtools (v1.2) [38] with default parameters. RNA editing sites were identified using the “sprint_from_bam” program of SPRINT [39] with default parameters.

### 2.2. Tissue-Specific Editing

To improve the accuracy of identification of tissue-specific editing sites, for each tissue, we removed the sites that were missing editing value measurements in more than one-third of the samples. The ROKU R package [40] was applied to rank the editing sites by their overall tissue specificity using Shannon entropy. All sites satisfy the requirements that the editing level range (maximum editing level minus minimum editing level) is larger than 0.1 and the Shannon entropy is less than 0.4 were reserved as tissue-specific editing sites. 

### 2.3. Sequence Preference 

Two Sample Logo [41] were used to show the enriched and depleted nucleotides nearby the A’s targeted for RNA editing (*p* < 0.01, *t*-test). Adenosine sites randomly chosen from reference were used as the negative control.

### 2.4. Genomic Feature Annotation

Human RNA editing sites were download from the RADAR database [28], then we adopted the UCSC LiftOver tool (https://genome.ucsc.edu/cgi-bin/hgLiftOver) to convert the genome position from human reference to pig reference. We also converted the genome position from pig reference to human reference. The RNA editing sites successfully converted on both turns (from the human to the pig and from the pig to the human) were reserved as conserved RNA editing sites. Pig quantitative trait locus (QTL) was downloaded from Pig QTLdb [42] and RNA editing sites located at QTL regions were selected using a python script.

### 2.5. Impacts of RNA Editing Sites on miRNA–mRNA Interactions

For each A-to-G editing site located at 3′UTR, we extracted the editing site and the flanking 25 bps from reference (in total 51 bp) as an unedited-type (UT) sequence and changed the editing site from A-to-G (or T-to-C) as an edited-type (ET) sequence. We then used RNAhybrid [43] and miRanda [44] to predict the miRNA target sites on UT and ET sequences. We generated four target datasets, which are RU (RNAhybrid predicted targets on UT sequence), MU (miRanda predicted targets on UT sequence), RE (RNAhybrid predicted targets on ET sequence), and ME (miRanda predicted targets on ET sequence). The miRNA–mRNA interactions that existed in both RU and MU, but in neither RE nor ME, were defined as interaction losses (loss = (RU and MU) – (RE or ME)). On the contrary, the miRNA–lncRNA interactions that existed in both RE and ME, but in neither RU nor MU, were defined as interaction gains (gain = (RE and ME) – (RU or MU)).

### 2.6. edQTL Analysis

Variant calling using Samtools (v1.2) was performed and we selected variant positions from each sample in which the mismatches were supported by at least two reads while at least 10 reads covering this site and both base and mapping quality scores were at least 20. For QTL mapping, we examined 630 RNA editing sites with editing level measured in at least one-third of the samples. For each of the 630 RNA editing sites we normalized the editing level using Z-score method across all samples. The following protocol was performed to map edQTLs genome-wide: (1) For each editing site, we fit linear models without any covariates between normalized editing levels and genotypes of each variant in the genome using Plink [45]. We only used variants in which the minor allele was present in at least 20 samples. We identified genome-wide edQTLs using a significance threshold of 2 · 10^−7^ (Bonferroni method).

### 2.7. Database Construction

PRESDB was built using Django Python Web framework (https://www.djangoproject.com) coupled with MySQL database. The front-end interface was developed based on Bootstrap open source toolkit (https://getbootstrap.com) and jQuery JavaScript library (https://jquery.com). PRESDB was published using Apache Http server and is accessible at https://presdb.deepomics.org.

## 3. Results

### 3.1. Identification and Profiling of *Sus scrofa* RNA Editing Sites

**Figure 1:**
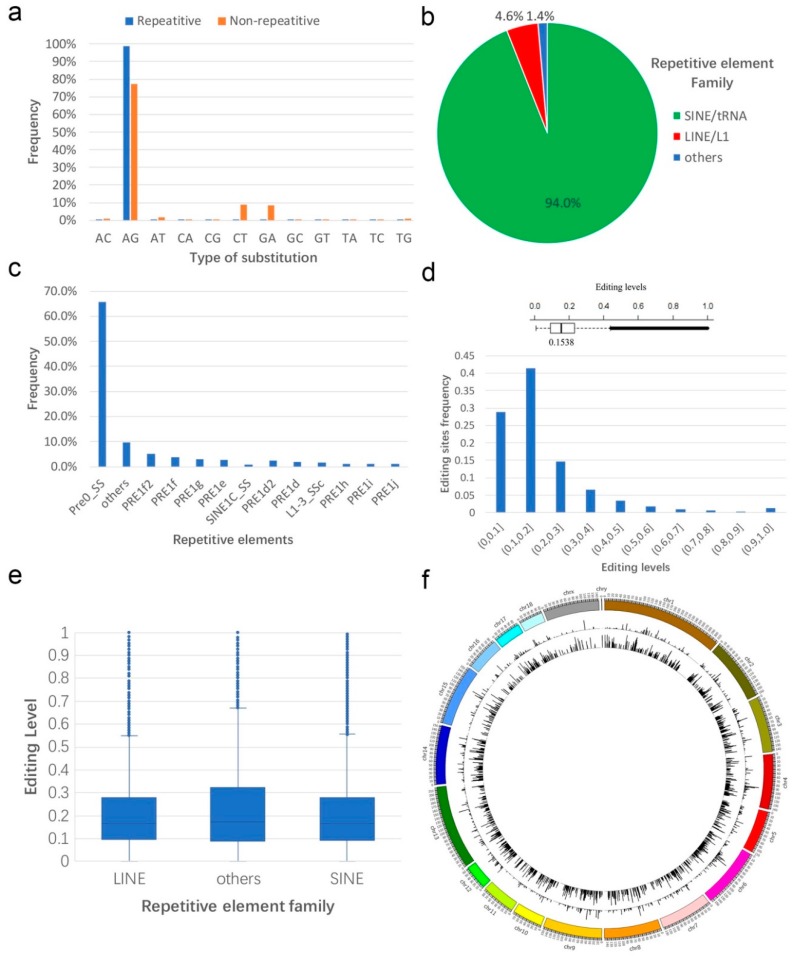
Identification and profiling of A-to-I RNA editing. (**a**) Percentage of 12 types of RNA editing events in repetitive and non-repetitive regions. (**b**) Distribution of repetitive A-to-G mismatches across major repetitive element families (**c**) and further broken down into specific repetitive element types. (**d**) Histogram and box plot showing the frequency of RNA editing levels. (**e**) Overall RNA editing levels across major repetitive element families. (**f**) Genome-wide distribution of RNA editing sites. From outside to inside, each circle represents the number of all editing sites and the average editing level of all RNA editing, respectively.

To identify RNA editing sites in *Sus scrofa*, we collected 211 RNA-seq samples across 11 tissues from the National Center for Biotechnology Information (NCBI). We performed reads mapping and RNA-editing sites identification with Histat2 and SPRINT (see Methods). The genome-wide screening of RNA editing yielded a total of over 490,000 RNA editing events in all eleven tissues. The percentage of A-to-G mismatch, which is consistent with A-to-I editing, was 94.6% in repetitive region and 77.4% in non-repetitive (Figure 1a). Because the primate Alu elements influenced RNA editing in both coding and non-coding regions [46], we restricted our search to swine repetitive sequences and searched the short interspersed nuclear elements (SINE) in pigs which were capable of attracting the majority of ADAR activity. The results showed that, similar to those reported in the pig [23], more than 94.0% (445,057 of the 473,412) A-to-G editing sites were within pig SINE elements as opposed to the long interspersed nuclear elements (LINE) (4.6%) and others (1.4%) (Figure 1b), although SINEs contributed 11.4% of the swine genome, while LINEs contributed 17.5%. Of the 445,057 repetitive A-to-G mismatches, 65.7% were found within the Pre0_SS element (Figure 1c), which was the most identical to the consensus PRE-1 sequence among all elements of the PRE-1 family.

The editing level of each site is calculated as the number of reads supporting this editing over the number of reads covering this locus. Similar to humans [47], rhesus macaque and flies [48], the editing level varied from 1% to 100% among different editing sites, with a median level of 15.38% in *Sus scrofa* (Figure 1d). Then, we investigated the difference of RNA editing level among repetitive element families. The result showed the median level of LINE, SINE, and others were 16.6%, 16.6%, and 17.3%, respectively (Figure 1e), although the SINE family account for the most A-to-G mismatches. In order to statistic RNA editing sites at the genome-wide level, each chromosome was split into contiguous 1-Mb windows from the beginning to the end, and the number of total RNA editing sites and average editing level within each window were calculated. The result showed that RNA editing events appeared on every chromosome without preference (Figure 1f), highlighting the pervasive nature of RNA editing. 

### 3.2. Annotation of RNA Editing Sites

**Figure 2:**
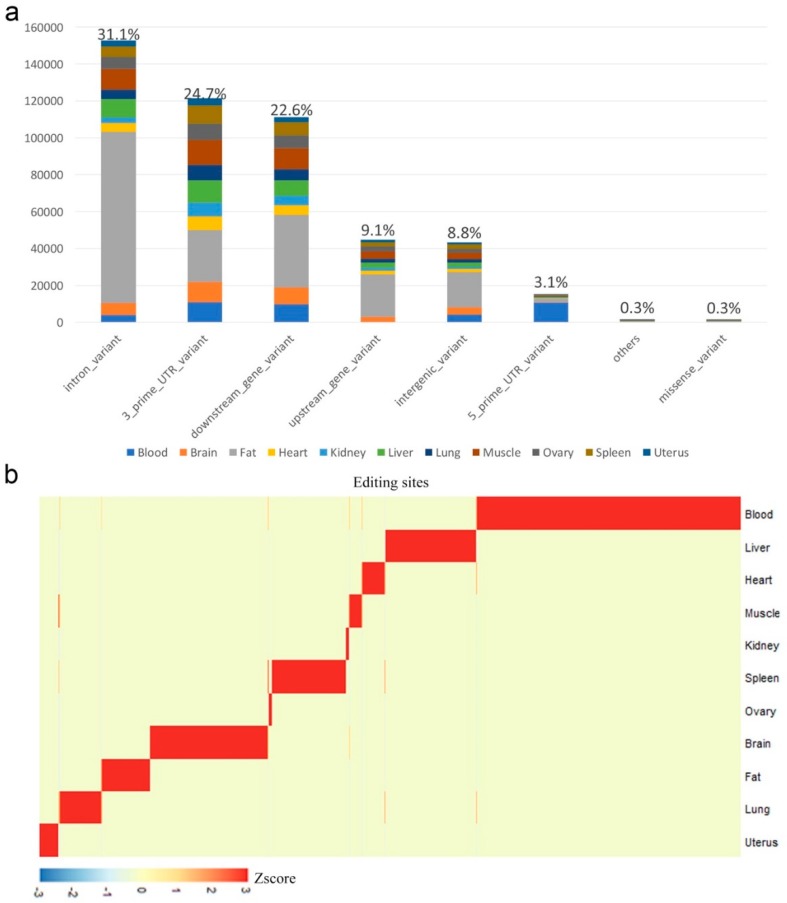
Annotation of RNA editing sites. (**a**) A-to-I mismatch locations relative to the nearest annotated gene. Percentages shown are out of all A-to-G mismatches. (**b**) Heat map of editing levels from sites that are specifically edited in one tissue.

To annotate the RNA editing sites, we used the SnpEff [49], a genomic variant annotations tool to find the genomic feature of each mismatch to the annotated gene. We annotated the mismatches according to eight genomic features: Intron variant, intergenic variant, downstream gene variant, upstream gene variant, missense variant, 3′-UTR variant, 5′-UTR variant, and others. Consistent with the previous study in pigs [23], 31.1% of all detected mismatches were located in retained introns (Figure 2a). The remaining sites were enriched in other non-coding regions including 3′ UTRs, intergenic, and gene proximal regions. According to our data, 980 pig mismatches within coding regions would result in a missense variant (Supplemental Table S1). Moreover, we identified tissue-specific RNA editing sites using Shannon entropy (see Methods), while the number of detected RNA editing events varied greatly among tissues (Figure 2b). Also, we compared with the human RNA editing sites using UCSC LiftOver tool (see Methods) and found 554 conserved RNA editing sites. Moreover, we compared these 554 conserved editing sites with the 59 previously published conserved mammalian sites [50] and found 13 common conserved editing sites (Supplemental Table S3). Then, we used a python script to find all RNA editing sites that were located in pig quantitative trait locus (QTL) regions. The results showed that about 14,238 RNA editing sites were located in the pig QTL regions where 53% and 17% of these sites were related with conformation score and front feet conformation, respectively (Table 1).

### 3.3. Sequence Preference of A-to-I Editing Sites

**Figure 3:**
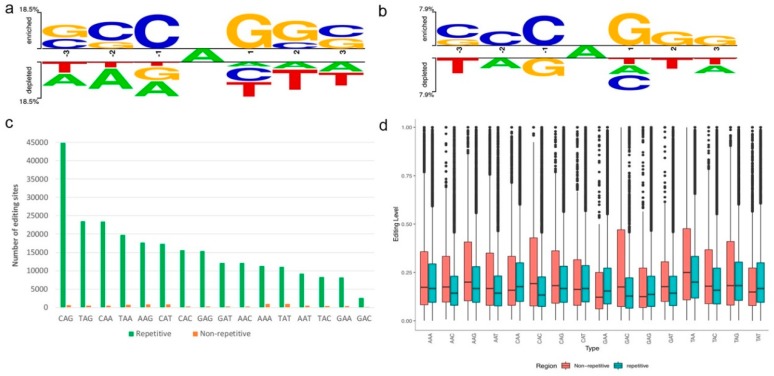
Sequence context of RNA editing sites. Sequence preferences for base positions flanking (−3, +3) detected A-to-I editing sites (**a**) in repetitive region and (**b**) non-repetitive region. (**c**) The number of 16 possible triplets centered on the edited adenosine (NAN). (**d**) Editing levels of editing sites in different triplets.

When investigating the flanking sequences of the A-to-I editing sites identified in this study, we observed C enrichment one nucleotide upstream (−1) and G enrichment one nucleotide downstream (+1) RNA editing sites located in the repetitive region (Figure 3a,c). Meanwhile, consistent with what has been found in human [48], RNA editing sites in the non-repetitive region showed similar significate characters as the repetitive region (Figure 3b). Also, in both repetitive and non-repetitive regions, we observed a weak base preference at the positions beyond the nearest neighboring nucleotides. Specifically, the −2 and +2 positions were slightly enriched for C and G, respectively. (Figure 3a,b). These findings suggest that A-to-I editing was influenced by more than the −1 and +1 nucleotides of the edited A’s. Our data showed that editing levels in non-repetitive regions varied more than these in repetitive regions (Figure 3d), which was similar with what has been reported in other mammals [39].

### 3.4. Impact of A-to-I Editing on miRNA–mRNA Interactions

**Figure 4:**
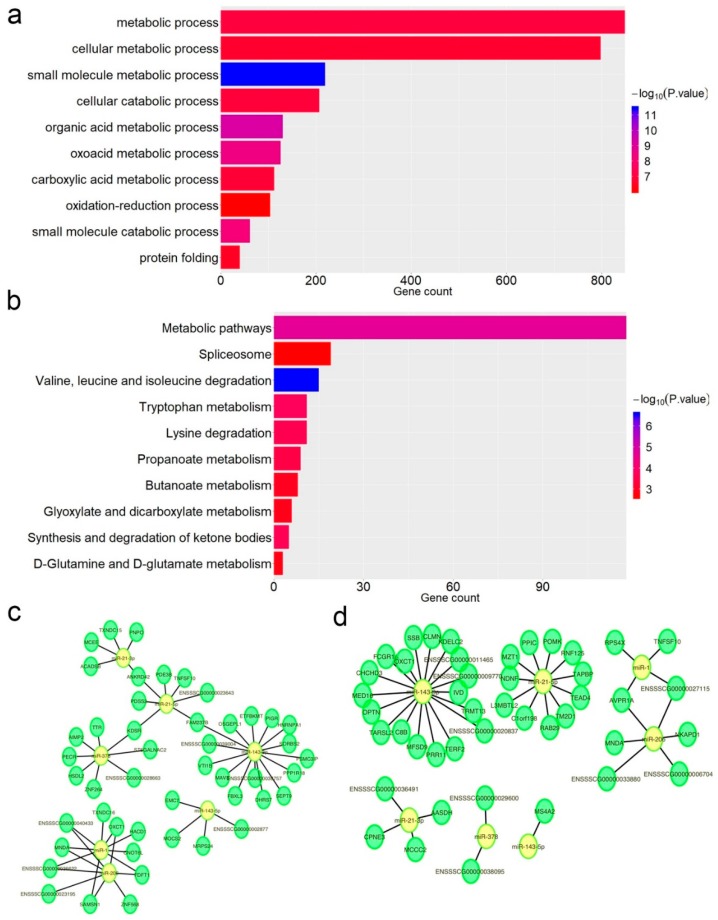
Impact of RNA editing on miRNA–mRNA interactions. (**a**) Gene Ontology (GO) analysis of all edited mRNAs. (**b**) Kyoto Encyclopedia of Genes and Genomes (KEGG) pathway analysis of all edited mRNAs. Network of (**c**) gain pairs or (**d**) loss pairs appeared with miR-1, miR-143, miRNA-206, miRNA-21 and miRNA-378.

Similar to Single Nucleotide Polymorphism sites (SNPs), editing sites also have the potential to influence the miRNA–mRNA interactions. In this work, we selected all A-to-G editing sites (38,272) that located at 3′UTR region and predicted miRNAs that can target on these editing sites using RNAhybrid and miRanda. After that, we identified editing sites potentially to disturb original (loss) miRNA target sites and/or to create new potential (gain) miRNA target sites (see Methods). The results showed that 4552 RNA editing sites disturbed 5189 miRNAs target sites and 4,176 RNA editing sites created 5729 new potential miRNA target sites. Together, we found 1525 mRNA were impacted, and gene ontology and Kyoto Encyclopedia of Genes and Genomes (KEGG) pathway analysis showed that biological function of these genes was enriched in metabolic pathways including small molecule metabolic process, organic acid metabolic process and cellular metabolic process (Figure 4a,b). Since pigs are primarily used to provide protein for humans, we selected several miRNAs that participated in skeletal muscle development including miR-1 [51], miR-143 [52], miRNA-206 [53], miRNA-21 [54] and miRNA-378 [55] and constructed the network of the changed miRNA–mRNA pairs caused by the A-to-G mismatches. The result provided nodes and connections between edited mRNAs and their target miRNAs. According to our data, more than one targets of these miRNAs were lost or gained after editing (Figure 4c,d). 

### 3.5. edQTL Analysis

**Figure 5:**
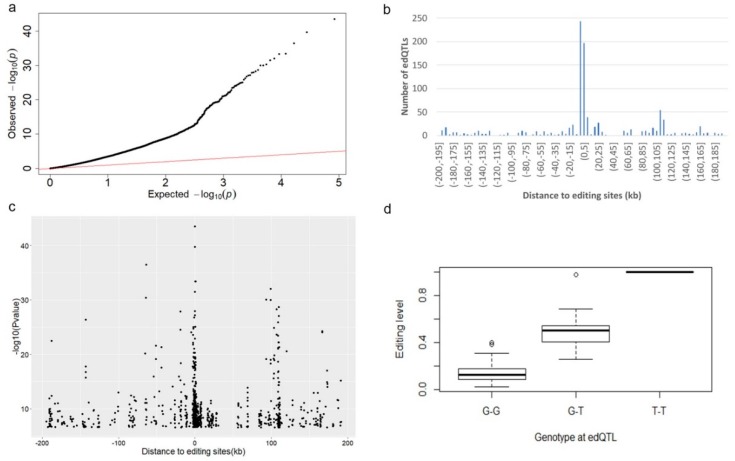
RNA editing quantitative trait loci (edQTLs) analysis. (**a**) Quantile–quantile (QQ) plot for association testing *p*-values of RNA levels with genetic variants within 200 kb of the 630 editing sites. (**b**) Statistic of the number of edQTLs located at different distance (kb) regions of the 630 RNA editing sites. (**c**) Significance of association tests in relation to the distance between the editing sites and variants. (**d**) Example of an edQTL. Box plot showing the association of edQTL (chr3: 102921373) with the editing level at chr3: 102985380.

Quantitative trait loci (QTL) mapping has been successfully applied to identify the regulatory mechanisms of many molecular phenotypes such as gene expression (eQTLs) [56] and splicing patterns (sQTLs) [49]. To identify genetic variants that could impact RNA editing levels, we ran association tests between editing levels of selected editing sites and genotypes for all variants (see Methods). In order to focus on cis-effects, we restricted the search space of RNA editing QTL (edQTL) between –200 kb to approximately 200 kb of the RNA editing site. We found most of the selected RNA editing events were associated with genomic polymorphisms (Figure 5a). Meanwhile, 21.7% of the selected RNA editing sites (137 of 630) were associated with more than one edQTL. As expected, edQTLs were highly enriched within 5 kb of their associated RNA editing site (Figure 5b). Moreover, we also observed there were a higher statistical significance and greater association with edQTLs that were closer to the RNA editing site (Figure 5c). An example of an RNA editing event that was associated strongly with a genetic polymorphism occured at chr3: 102, 985, 380 where the T-allele is associated with a high level of RNA editing while the G-allele nearly abolishes RNA editing (Figure 5d).

### 3.6. *Sus scrofa* RNA Editing Sites Database

**Figure 6:**
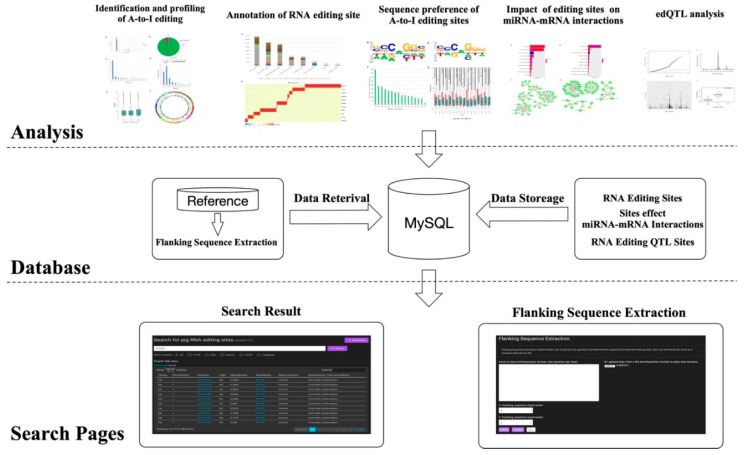
Framework of the database construction in PRESDB.

In order to provide users an easy access to pig RNA editing data, we built a database of pig RNA editing sites (PRESDB), containing more than 490,000 RNA editing sites across 11 tissues. PRESDB has four functional pages: An RNA editing site search page, a flanking sequence extraction page, a miRNA–mRNA interaction page, and an RNA editing QTL (edQTL) page (Figure 6). The search page allows users to search interested RNA editing sites by giving a gene name (e.g., PLPPR1) or a genome region (e.g., 1:271477204-271479179). The search result table contains 10 information columns: Tissue, chromosome, position, editing type, editing level, gene name, gene location, quantitative trait locus (QTL), conserved in human, and tissue-specific. Users can click position column to view one specific editing sites in the UCSC genome browser or click the gene name column to view the specific function of this gene on the GeneCards website. The flanking sequence extraction page provides the user with the ability to extract upstream and downstream sequences of the interested RNA editing sites to perform some downstream analysis (e.g., ADAR-binding sequence preferences). The extracted sequences can be downloaded in standard FASTA format. A data table is used to display all editing sites with the potential to disturb original miRNA target sites or to create new potential miRNA target sites for each tissue in the miRNA–mRNA interaction page. The edQTL page collects all RNA editing QTL (edQTL) sites within 200 kb of the RNA editing sites. To facilitate more detailed searches, we have made the entire database contents available as CSV files on the web page. 

## 4. Discussion

Recently, an increasing number of A-to-I RNA editing sites have been published in mice and human. As RNA editing sites appeared in a tissue-specific manner, it is essential that studies of RNA editing in mammals assess various tissues. The pig is not only an important farm animal that provides protein for humans but an important non-rodent animal model that is widely used in biomedical research. Thus, to perform genome-wide identification of *Sus scrofa* RNA editing sites and explore their patterns, we collected RNA sequencing data across 11 tissues (blood, brain, fat, heart, kidney, liver, lung, muscle, ovary, spleen, and uterus). We identified more than 490,000 *Sus scrofa* RNA editing sites, which represent a significant addition to the growing catalog of annotated mammalian RNA editing sites. Moreover, similar to results that have been reported in human and mouse RNA [47], *Sus scrofa* RNA editing sites showed high enrichment in repeated regions and low editing level. Our data indicated that the pig transcriptomes are highly editable among PRE-1 SINE retrotransposons which have similar features to the primate Alu. Since Alu elements enable substantial RNA editing among primate genomes, RNA editing in pigs may be achieved through a similar mechanism.

Consistent with previous studies, our data showed that the majority of RNA editing sites are located in non-coding regions, including intron, intergenic, and gene proximal regions, while little is known about the mechanism of the average effect of RNA editing in these non-coding regions. An instance in human RNA demonstrated that intronic A-to-G editing events contribute to alternative splicing of nuclear prelamin A. Thus, these editing sites are useful to understand the extent to which this phenomenon enhances pig genetic variation. Furthermore, we also identified 980 RNA editing sites that could result in missense mutation in 560 genes. Among these 560 genes, GRIA2 and GRIK2, both found to be edited in brain tissue, were previously identified as genes edited during porcine neural tissue development. ACOX1, CPT1B, CPT2, CYP27A1, and ACSL1, etc. participate in Peroxisome proliferator-activated receptor (PPAR) signaling pathway which plays a critical physiological role in the regulation of numerous biological processes, including lipid and glucose metabolism, and overall energy homeostasis. Therefore, these missense mutations may play important roles in the development and energy metabolism of the pig.

Although the molecular functions of RNA editing sites are mostly unclear, functional consequences of A-to-I RNA editing sites in coding and non-coding regions have been found, including synonymous or nonsynonymous within many RNAs, miRNA biogenesis, and editing of miRNA seed regions. To assess whether *Sus scrofa* RNA editing sites affect miRNA and mRNA interactions, we predicted miRNA-originating targets and miRNA-edited targets by two computational methods. Moreover, we found editing sites with the potential to disturb original miRNA target sites and to create new potential miRNA target sites. The function of these target genes was enriched in metabolic pathways including a small molecule metabolic process and an organic acid metabolic process, indicating the importance of RNA editing events in metabolic processes. Moreover, as most RNA editing sites reside in intergenic and intron regions, RNA editing sites are considered to have impacts on lncRNA secondary structures. Because of the incomplete annotation of lncRNA in the *Sus scrofa* reference, we did not explore the effects of RNA editing sites on lncRNA secondary structure, which can be done in a future study. 

In order to investigate the cis variation of RNA editing in *Sus scrofa*, we tested the correlations between RNA editing sites and variants within 200 kb of the RNA editing site. We observed that variants within 1 kb of editing sites were more likely to have significant associations. Therefore, in a future study, these edQTLs can be used to bridge the gap in our understanding between RNA editing level and their respective susceptibility loci. Although edQTLs have been proved to affect editing levels through changes in the local secondary structure for edited dsRNA in human beings, the mechanism by which edQTLs affect editing levels in the pig remain to be studied.

At present, RNA editing sites have been discovered in plants, animals, and humans, but are rare in *Sus scrofa*. Exploring the pig genome can illuminate facets of human culture, organic evolution, biomedical research, and animal breeding. Therefore, we constructed the database of *Sus scrofa* RNA editing sites based on our data. To the best of our knowledge, this database is the first public resource containing information about the RNA editing sites of *Sus scrofa*. Although these identified editing sites in our database, especially in the coding regions, remain to be experimentally validated, our database will enhance pig genetic variation and provide explanations for a variety of traits of interest to both biomedical and agricultural communities.

## Figures and Tables

**Table 1:** Statistic of RNA editing events in QTL regions.

Quantitative Trait Locus (QTL)	Number	Percentage
Conformation score	7536	53.01%
Front feet conformation	2431	17.10%
Maternal infanticide	795	5.59%
Ear weight	604	4.25%
Rhinitis	569	4.00%
Hind leg conformation	475	3.34%
Vertebra number	442	3.11%
Spinal curvature	422	2.97%
Left teat number	414	2.91%
Lumbar vertebra number	257	1.81%
Time spent feeding	188	1.32%
Ear erectness	54	0.38%
Ear size	15	0.11%
Umbilical hernia	11	0.08%
Thoracic vertebra number	2	0.01%

## Supplementary Materials

The following are available online at https://www.mdpi.com/2073-4425/10/5/327/s1. Table S1: RNA editing sites resulting in amino acid changes. Table S2: Summary of tissue samples for total RNA sequencing in *Sus scrofa*. Table S3: List of 13 common conserved editing sites.
