# Genome-Wide Investigation and Functional Analysis of *Sus scrofa* RNA Editing Sites across Eleven Tissues

**DOI:** 10.3390/genes10070520

**Published:** 2019-07-10

**Authors:** Zishuai Wang, Xikang Feng, Zhonglin Tang, Shuai Cheng Li

**Affiliations:** 1 Department of Computer Science, City University of Hong Kong, Kowloon, Hong Kong; zishuwang2-c@my.cityu.edu.hk (Z.W.); xikanfeng2-c@my.cityu.edu.hk (X.F.); 2 Department of Pig Genomic Design and Breeding, Agricultural Genome Institute at Shenzhen, Chinese Academy of Agricultural Sciences, Shenzhen 518124, China

Two errors occurred in the References part of our paper [1]: One for the reference of SPRINT software in Section 2.1, and another for the reference of SnpEff software in Section 3.2. The correct reference for SPRINT is “SPRINT: An SNP-free toolkit for identifying RNA editing sites” [2] and the correct reference for SnpEff is “A program for annotating and predicting the effects of single nucleotide polymorphisms, SnpEff: SNPs in the genome of Drosophila melanogaster strain w1118; iso-2; iso-3” [3]. In addition, the supplementary tables in our paper [1] are not cited in order. The correct order has been updated and the supplementary Tables S1, S2 and S3 has been renamed as Tables S1, S2 and S3 without including “supplementary” before.

We would like to apologize for any inconvenience caused. The change does not affect the scientific results.

## Supplementary Materials

Supplementary File 1
